# Supplementary material for: Naive human B cells engage the receptor binding domain of SARS-CoV-2, variants of concern, and related sarbecoviruses
Source: Sci Immunol. Author manuscript; Available in PMC 2022 Dec 10. (PMC8720485; doi:10.1126/sciimmunol.abl5842)
Supplement: Supplmental Text — Figure S1. Design and characterization of SARS-CoV-2 antigens and healthy donor sera binding. Figure S2. PBMC flow cytometry analyses. Figure S3. Repertoire comparison, germline identity, and IgG binding by individual donor. Figure S4. SARS-CoV-2 RBD-binding kinetics of isolated naive antibodies. Figure S5. Structural characterization and analysis of ab090. Figure S6. Representative affinity maturation selection strategy and output sequence overview. [file NIHMS1765250-supplement-Supplmental_Text.docx]

**Supplemental Information**

**Naive human B cells engage the receptor binding domain of SARS-CoV-2, variants of concern, and related sarbecoviruses**

Jared Feldman^1†^, Julia Bals^1†^, Clara G. Altomare^2^, Kerri St. Denis^1^, Evan C. Lam^1^, Blake M. Hauser^1^, Larance Ronsard^1^, Maya Sangesland^1^, Thalia Bracamonte Moreno^1^, Vintus Okonkwo^1^, Nathania Hartojo^1^ Alejandro B. Balazs^1^, Goran Bajic^2^, Daniel Lingwood^1*^ and Aaron G. Schmidt^1,3*^

^1^Ragon Institute of MGH, MIT and Harvard, Cambridge, MA, 02139, USA

^2^ Department of Microbiology, Icahn School of Medicine at Mount Sinai, New York, NY 10029

^3^Department of Microbiology, Harvard Medical School, Boston, MA 02115, USA

*Correspondence to: Email: dlingwood@mgh.harvard.edu (D.L.); aschmidt@crystal.harvard.edu (A.G.S)

†These authors contributed equally to this work.

**This PDF file includes:**

**Figs. S1 to S6**

**
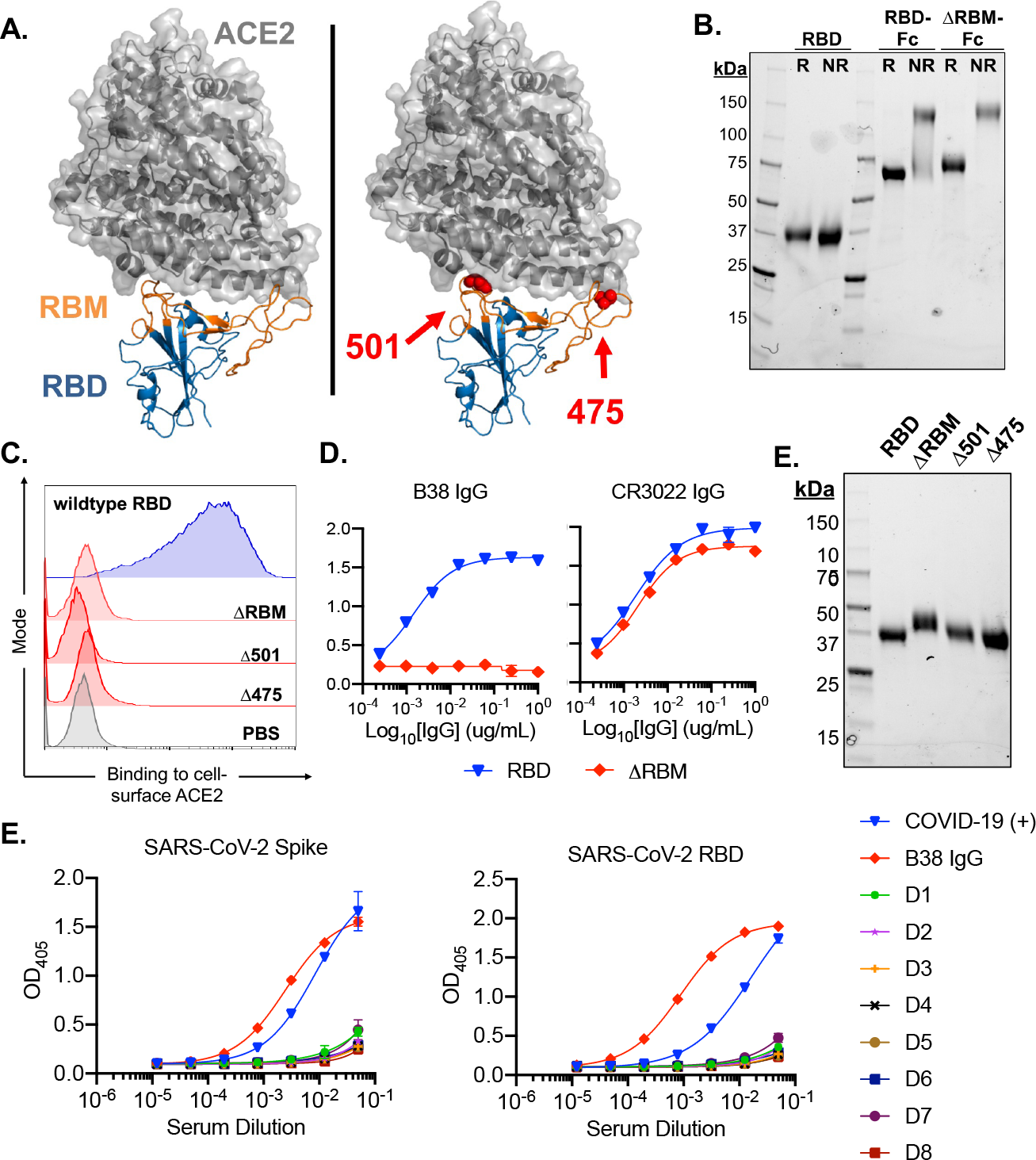
**

**fig. S1. Design and characterization of SARS-CoV-2 antigens and healthy donor sera binding.** **(**A) SARS-CoV-2 RBD in complex with viral receptor, ACE2 shown in blue and grey, respectively (PDB 6M0J). Wild-type RBD with, the receptor binding motif (RBM), shown in orange (left panel). Structural model of the ∆RBM probe designed to abrogate binding to ACE2 (right panel). Putative N-linked glycosylation sites engineered onto the RBM are shown in red spheres at amino acid positions 501 and 475. (**B**) SDS-PAGE gel under reducing (R) and non-reducing (NR) conditions for monomeric RBD, RBD-Fc and ∆RBM-Fc. (**C**) Wildtype RBD, ∆RBM and single glycan variant binding to ACE2-expressing 293T cells by flow cytometry. Wild-type RBD binding shown in blue, glycan variant binding shown in red. Streptavidin-PE was used to detect the relative intensity of antigen binding to cell-surface ACE2. A PBS control (gray) indicates secondary-only staining. (**D**) Control antibody ELISA binding to RBD and ∆RBM antigens. RBM-specific antibody, B38 (left). Non-RBM-specific control antibody, CR3022 (right). (**E**) ∆RBM and ∆501 and ∆475 variants analyzed by SDS-PAGE gel under reducing conditions; wildtype RBD is shown for comparison. (E) SARS-CoV-2 spike (left) and RBD (right) sera ELISA from human subjects 1-8. Sera from a COVID-19 convalescent patient and control antibody, B38, were included as positive controls.

**
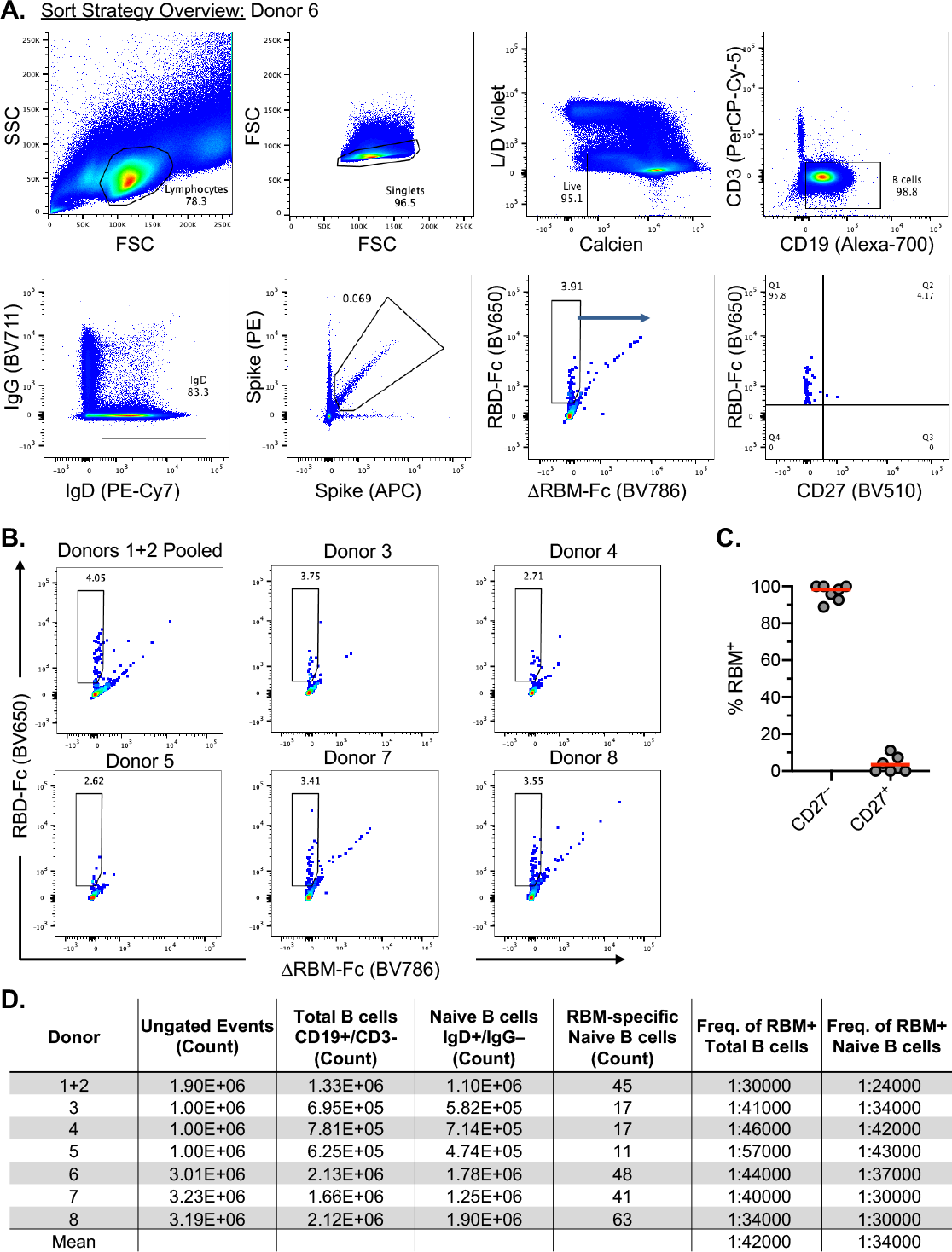
**

**fig. S2**. **PBMC flow cytometry analyses.** (**A**) Representative gating strategy used for FACS of PBMCs pooled from donor 6. Gating was on naive B cells defined by single living lymphocytes that were CD19^+^CD3^-^IgD^+^IgG^-^. Sorted cells were RBM-specific as defined by spike-PE^+^/spike-APC^+^/RBD-Fc-BV650^+^/∆RBM-Fc-BC650-. Sort gate is denoted by the blue arrow. The bottom right plot shows CD27 staining of sorted RBM-specific naive B cells. (**B**) Flow cytometry showing the sort gate and percentage of RBM-specific B cells for the remaining 7 healthy human donors with donors 1 and 2 pooled. (**C**) RBM-specific B cell frequency among CD27^+^ and CD27^-^ cells. Each symbol represents a different donor (*n* = 8). (**D**) Summary of events collected across all seronegative donors including frequency of RBM-specific events among total and naive B cell subsets.

**
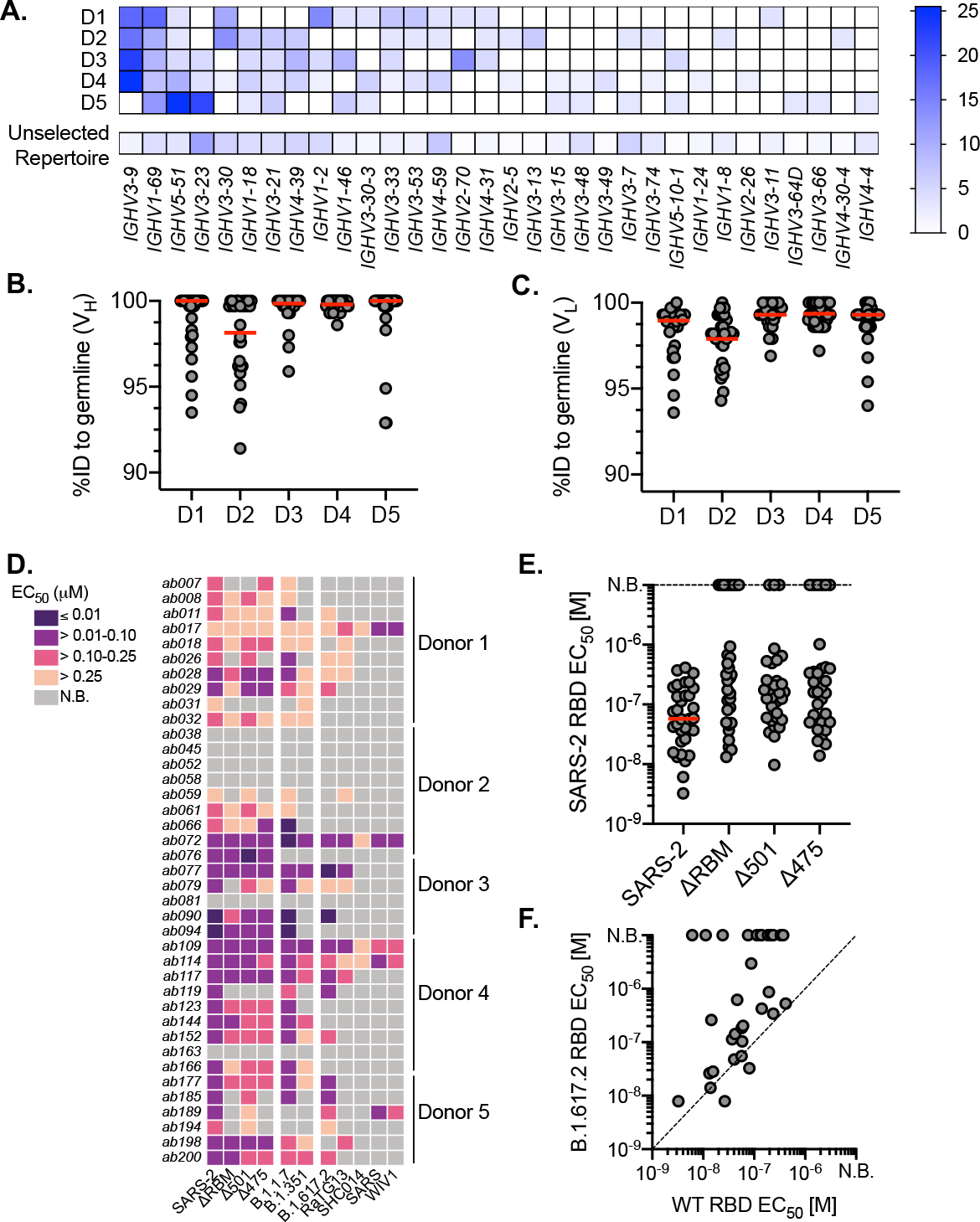
**

**fig. S3**. **Repertoire comparison, germline identity, and IgG binding by individual donor**. (**A**) Heatmap showing V_H_-gene usage of isolated antibodies derived from donors 1-5. Unselected repertoire gene usage derives from a high-throughput sequencing data set of circulating B cells across 10 human subjects (*23*). Heatmap scale represents percent of total paired sequence from each donor. Divergence from inferred germline gene sequences separated by individual donor for (**B**) V_H_ and (**C**) V_L_. Red bars indicate the median percent values, and each dot represents an individual paired sequence. (**D**) Heatmap showing IgG binding to RBDs (*n* = 38) sorted by donor. **(E**) ELISA EC_50_ values for IgGs with detectable SARS-CoV-2 RBD binding (*n* = 33) against RBM glycan probes. Red bars indicate the mean EC_50_ values.

**
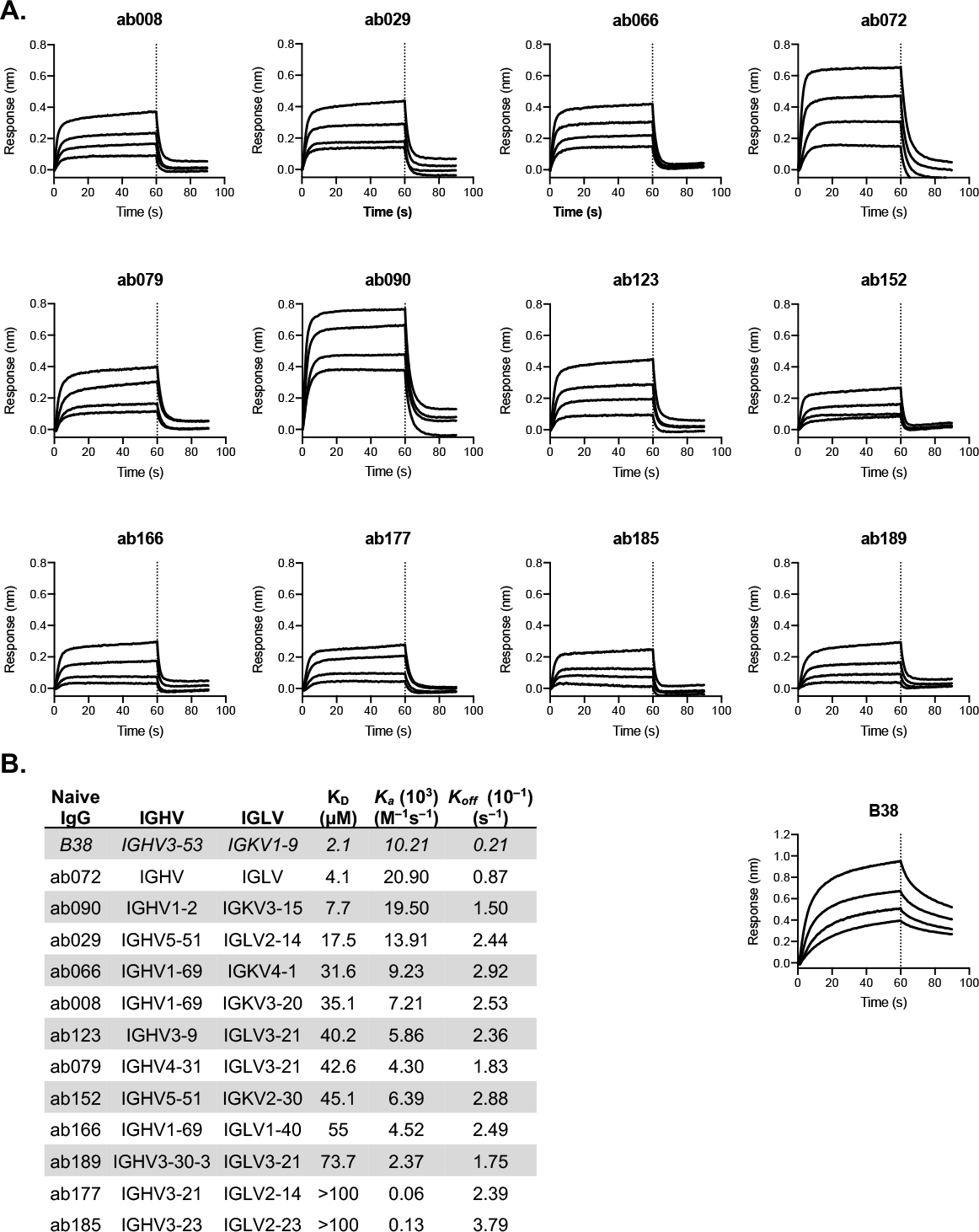
**

**fig. S4**. **SARS-CoV-2 RBD-binding kinetics of isolated naive antibodies.** (**A**) Biolayer interferometry (BLI) binding kinetic analysis of titrated SARS-CoV-2 RBD to immobilized Fabs. Dotted line at 60 s denotes the start of the dissociation phase. (**B**) Kinetic and equilibrium constants for binding to RBD calculated from a 1:1 binding model using a global fit to all curves for each Fab using vendor supplied software. B38 Fab is used as a positive control.

**A.**

**
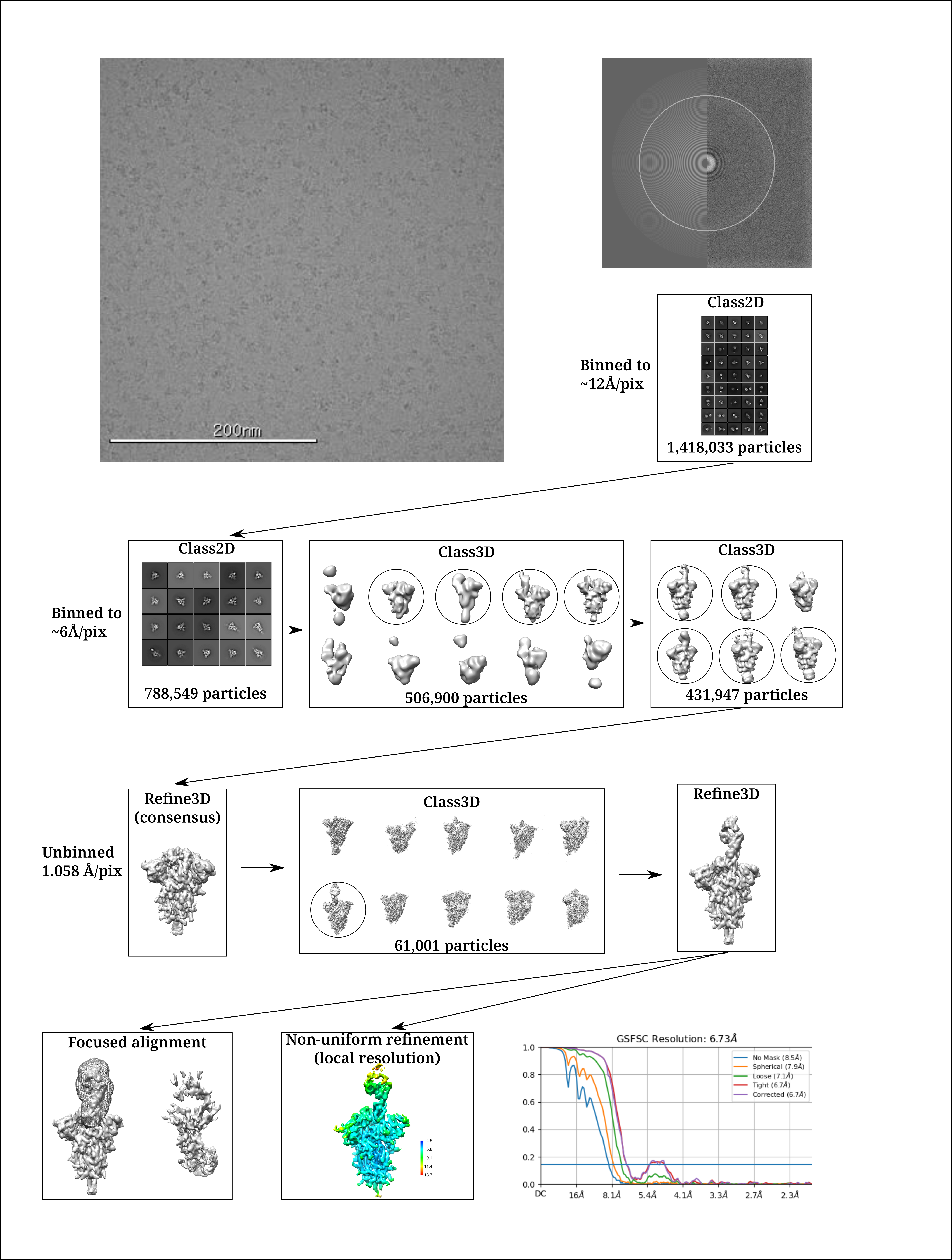
**

**B.**


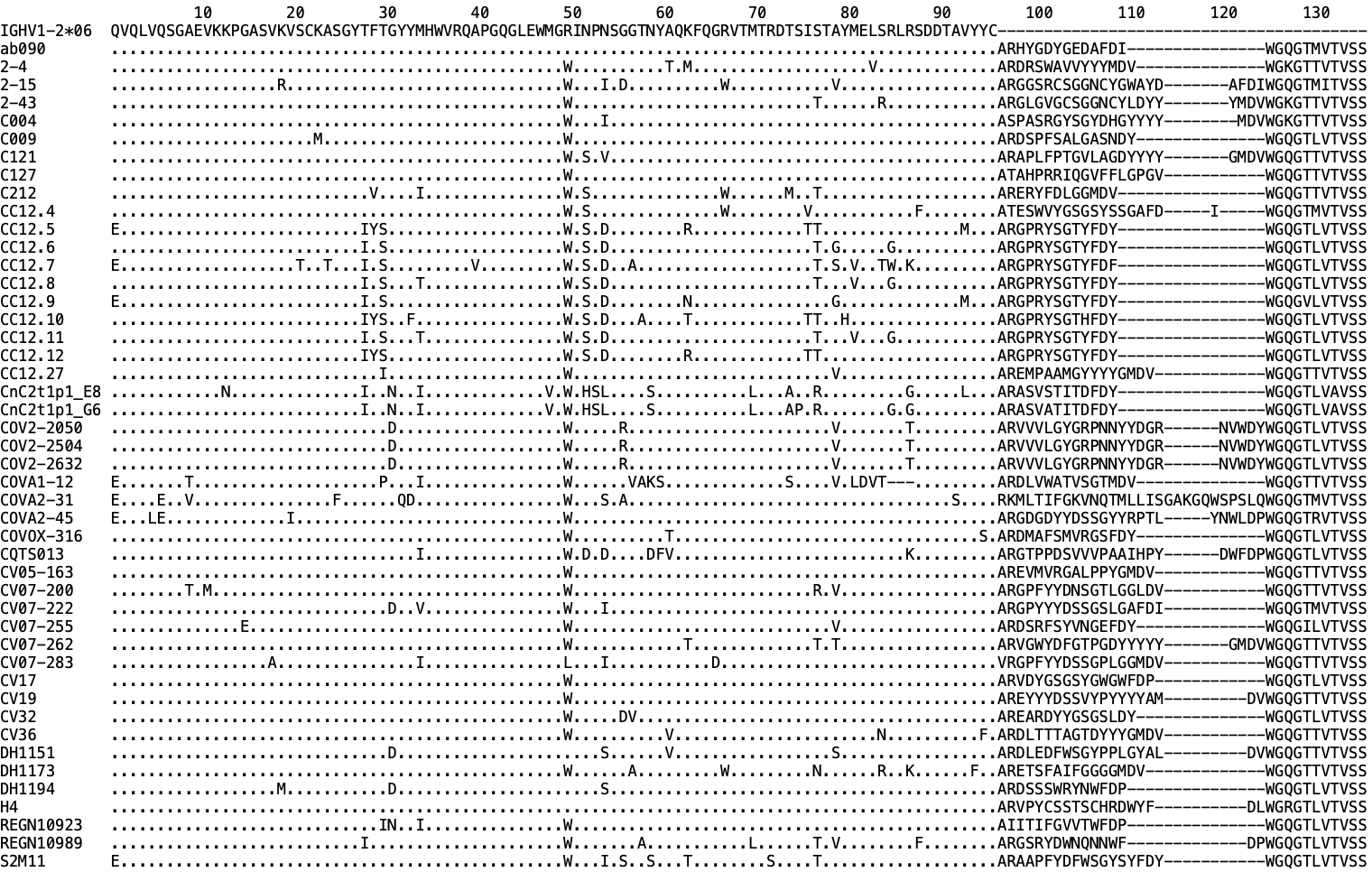


**fig. S5. Structural characterization and analysis of ab090. (**A**)** Cryo-EM data processing scheme of ab090 Fab bound with SARS-CoV-2 spike. See the Methods section for more details. (B) Sequence alignment of known IGHV1-2 antibodies.

**
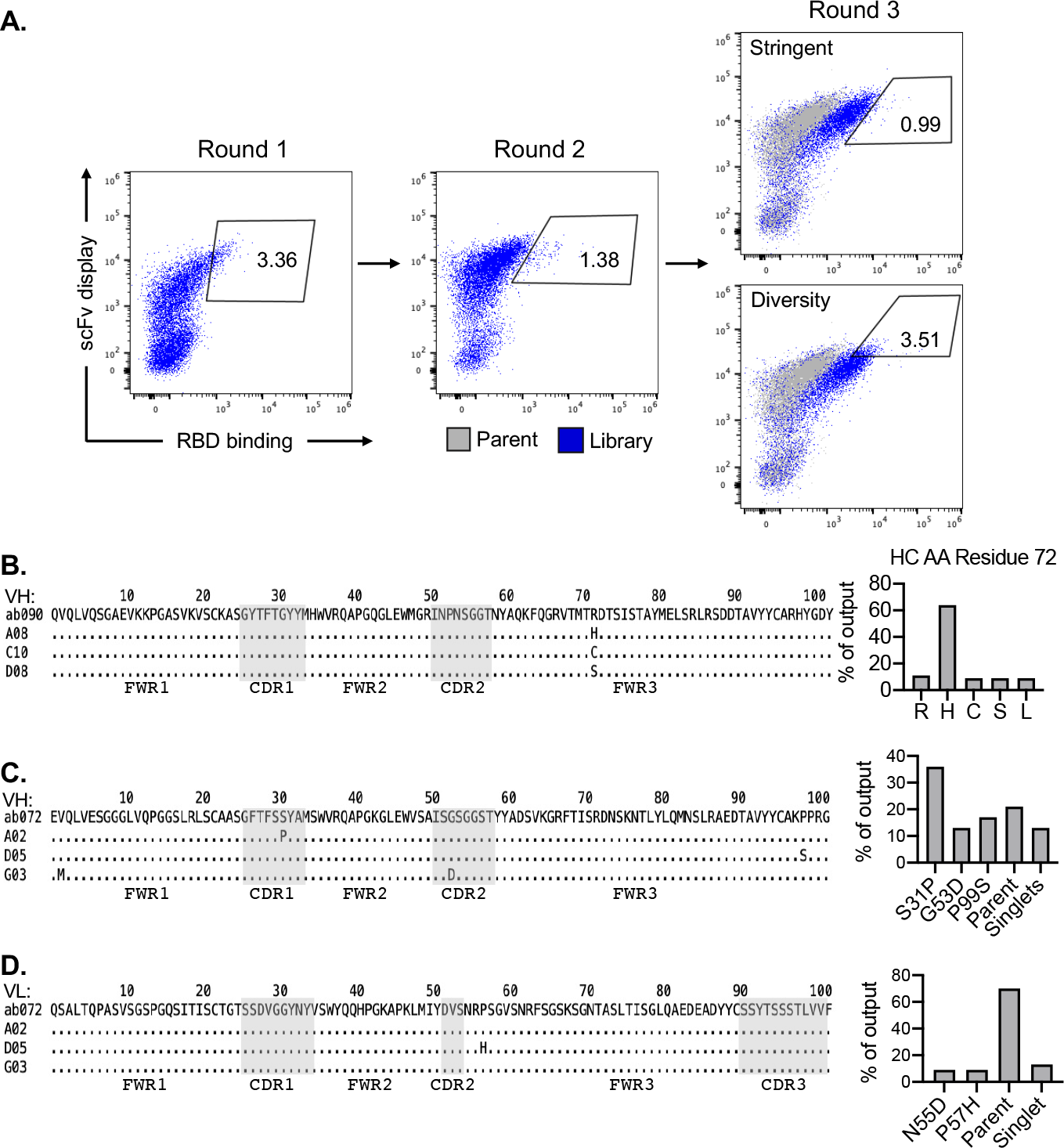
**

**fig. S6. Representative affinity maturation selection strategy and output sequence overview.** (**A**) Flow cytometric sorting of diversified single chain variable fragment (scFv) libraries of ab090. Gates represent the yeast population sorted for subsequent selections. After 2 rounds of enrichment for wildtype SARS-CoV-2 binding, “stringent” and “diversity gates were sorted in round 3 indicating the yeast populations sorted for individual colony isolation and sequencing. Alignment of the V_H_ sequencing output clones for ab090 (**B**) and ab072 (**C**) with the output frequency of each mutation from a total of 48 single colonies. (**D**) Alignment of the V_L_ sequencing output clones ab072 with the output frequency of each mutation from a total of 48 single colonies. The V_L_ output for ab090 was exclusively parent.
